# Natural Product Discovery Using Planes of Principal Component Analysis in R (PoPCAR)

**DOI:** 10.3390/metabo7030034

**Published:** 2017-07-13

**Authors:** Shaurya Chanana, Chris S. Thomas, Doug R. Braun, Yanpeng Hou, Thomas P. Wyche, Tim S. Bugni

**Affiliations:** 1 Pharmaceutical Sciences Division, School of Pharmacy, University of Wisconsin, Madison, WI 53705, USA; schanana@wisc.edu (S.C.); csthomas4@wisc.edu (C.S.T.); drbraun1@wisc.edu (D.R.B.); yanpenghou@gmail.com (Y.H.); thomas.wyche@merck.com (T.P.W.); 2 Exploratory Science Center, Merck & Co., 320 Bent St., Cambridge, MA 02141, USA

**Keywords:** metabolomics, principal component analysis, actinobacteria, marine actinomycetes, mass spectrometry

## Abstract

Rediscovery of known natural products hinders the discovery of new, unique scaffolds. Efforts have mostly focused on streamlining the determination of what compounds are known vs. unknown (dereplication), but an alternative strategy is to focus on what is different. Utilizing statistics and assuming that common actinobacterial metabolites are likely known, focus can be shifted away from dereplication and towards discovery. LC-MS-based principal component analysis (PCA) provides a perfect tool to distinguish unique vs. common metabolites, but the variability inherent within natural products leads to datasets that do not fit ideal standards. To simplify the analysis of PCA models, we developed a script that identifies only those masses or molecules that are unique to each strain within a group, thereby greatly reducing the number of data points to be inspected manually. Since the script is written in R, it facilitates integration with other metabolomics workflows and supports automated mass matching to databases such as Antibase.

## 1. Introduction

While natural products have provided important drug leads, especially in the area of oncology and infectious disease, the abundance of known molecules plagues the discovery process. In the case of actinomycetes and antibiotic discovery, it is well known that traditional methods of studying soil-based actinomycetes for antibiotics predominantly leads to the discovery of known classes. In general, the discovery challenge is not the lack of new molecules, but the abundance of known molecules. While actinomycetes from non-traditional niches such as insect symbionts [1,2,3], caves [4], coal-mines [5,6,7], deep sea sediments [8,9,10], and marine invertebrates [11,12,13,14] have increased the rate of the discovery of new scaffolds, the discovery of known compounds continues to present a critical barrier that limits the discovery pipeline. To overcome this critical barrier, new methods are needed that help identify the most promising bacterial strains. Whole genome sequencing holds great promise, but is still limited due to the resources required for broadly applying genome sequencing for strain prioritization. Mass spectrometry-based methods offer rapid, sensitive, and low-cost routes for strain prioritization. We and others have demonstrated that multivariate analysis of liquid chromatography-mass spectrometry (LC-MS) traces can be used effectively for strain prioritization and discovery [15,16,17,18,19,20,21,22,23,24]. In particular, principal component analysis (PCA) has been effective for the identification of strains producing putative novel natural products.

In general, the occurrence of novel molecules in actinomycetes is relatively rare compared to identifying a known molecule. Genomic insights have also shown that within certain groups of actinomycetes there often exists a core set of known biosynthetic clusters with randomly distributed clusters that likely encode for a novel natural product. The ability to identify molecules that are rare or unique within a collection of actinomycetes, however, can be difficult. While LC-MS provides a means to analyze the products, the inherent sensitivity of modern mass spectrometry leads to highly complex datasets. Additionally, the ionization efficiency of “interesting” molecules is not known a priori. So, methods that can identify molecules unique to particular strains offer a route to reduce the number of *m*/*z* values that need to be inspected as potential novel natural products. We have demonstrated that finding so-called “outliers” using PCA has been an effective route for the discovery of novel natural products [2,14,25,26].

In addition to our studies, two studies based on genomics have also provided justification for providing tools that could easily identify metabolites unique to strains in a study. Metcalf and Kelleher analyzed 830 actinobacterial genomes generating gene cluster families (GCFs) that provided some insight into the distribution of cluster families that equate to families of natural products [27]. Additionally, Paul Jensen’s group published a key paper that surveyed the genomes of the marine actinomycete genus, *Salinispora* [28,29]. Taken together, the data from these papers indicated that the most unusual gene clusters tend to be rare in occurrence while some classes are common. For example, Metcalf and Kelleher showed that related strains shared 80% of their nonribosomal peptide synthetases (NRPSs) and 73% of their type II polyketide synthase (PKS) GCFs [27]. Similarly, Jensen’s group showed that there were a core set of metabolites within each of the three species of *Salinispora* while the more unusual biosynthetic clusters were more random in occurrence [28,29]. Combined with our observations that identifying “unique” molecules within related groups of actinomycetes leads to the discovery of novel chemotypes, there is a commonality among observations that suggests that looking only at molecules unique to strains would be an advantageous path for quickly identifying putative novel natural products. While conceptually simple, a major issue arises when nothing is known about the genomes or chemistry of a collection of strains. PCA, at least in theory, is well suited to provide a map of molecules that are unique to strains within a collection. Challenges arise, however, from the fact that PCA is based in statistics and, when applied to LC-MS data, is typically biased toward molecules that ionize well. Unique molecules that ionize poorly tend not to have a large statistical influence on the data. Nonetheless, poorly ionizing molecules or those of low abundance can be found in higher principal component (PC) planes that from a statistical standpoint have little impact on the overall variance of the model. In fact, we have found that analyzing higher PC planes can lead to the discovery of novel molecules.

Given the complexities of applying LC-MS-PCA as an untargeted discovery tool for actinomycetes, we sought to develop scripts in R that would take LC-MS-based peak tables, generate a PCA model, and then search the model to identify planes where molecules unique to a strain could be identified. Then, the unique features (*m*/*z*-retention time) are automatically exported to a CSV file for inspection. Additionally, we enabled features to facilitate automatic dereplication processes. In our case, we purchased Antibase and were able to integrate Antibase into the workflow. However, other databases can be easily interfaced with the workflow. It is important to stress that by employing PCA, the script greatly reduces the number of data points that need to be inspected manually. By focusing on the differences between strains, it allows us to quickly look at the outlying data for unique molecules. This facilitates easier dereplication and can also be used to prioritize strains prior to extract production, for whole genome sequencing, or application of MSMS approaches such as Global Natural Products Social Molecular Networking (GNPS) [30]. In addition to marine natural products, the script can be integrated into other metabolomics workflows such as plant or NMR metabolomics with minor modifications.

## 2. Results

### 2.1. Untargeted Metabolomics and Feature Analysis

**Figure 1:**
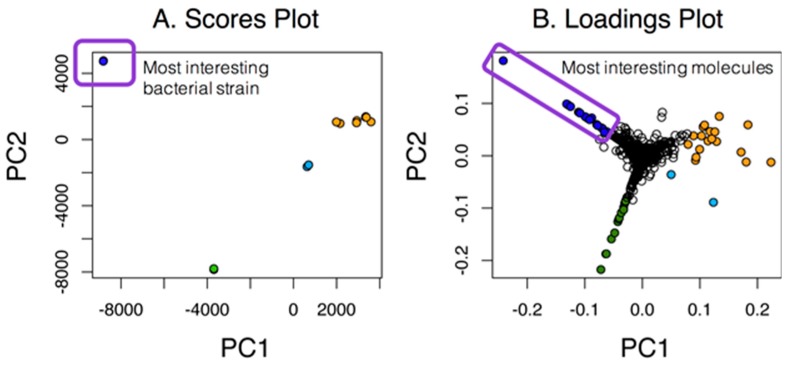
A PCA scores and loadings plot for seven marine actinomycetes with two replicates. For this case, the strain represented by the dark blue circles in the scores plot was the most unique, as indicated by the large separation in PC1. Molecules that were unique to that strain were color-coded in the same dark blue in the loadings plot.

Our first goal was to assess the number of unique molecular features within datasets as compared to the overall number of features. As highlighted in Figure 1, simple cases allow the inspection of PC1 vs. PC2, and in this case, the most unique strain is easily identified along with the most interesting molecules from the associated loadings plot. This simple case shows the relationship among strains and molecules in the loadings plot that were unique to either one particular strain or groups of strains (in orange). Unfortunately, many PCA scores plot are not as clear cut and would require additional analyses of higher PC planes.

**Figure 2:**
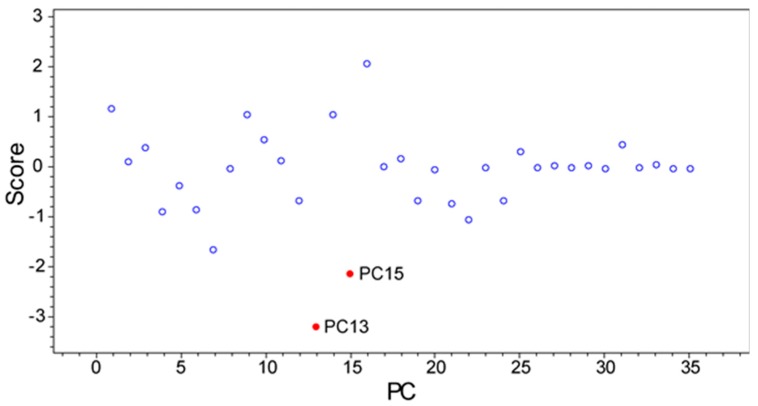
A scores overview plot for strain WMMB-499 in dataset one. To view the unique molecules associated with WMMB-499, PC13 vs. PC15 would provide the greatest separation from the other strains in this analysis because the absolute value of the score difference is the greatest.

**Figure 3:**
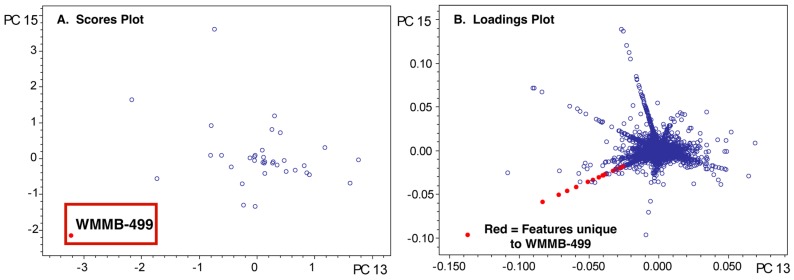
(**A**) The scores plot for PC15 vs. PC13 shows WMMB-499 separate from the other strains; (**B**) The loadings plot shows molecular features that are unique for WMMB-499.

To highlight aspects of feature finding and PCA, we chose two datasets where we identified unique features and then isolated compounds associated with those features. For example, we published two classes of novel compounds from an *Actinomadura* sp. (Strain WMMB-499) and patented a third [14,31]. The molecular features that suggested WMMB-499 was unique were identified in PC planes (PC13 vs. PC15) that would normally be considered insignificant from a statistical perspective. Nonetheless, many of the interesting molecules had low ion intensity, often below the level necessary for data-dependent MSMS. Depending on the scaling and dataset, unique aspects of a particular strain can only be identified in higher PC planes. We discovered the unique features for WMMB-499 by surveying planes manually and subsequently using Bruker Profile Analysis via a Scores overview plot to re-evaluate (Figure 2). While a Scores overview is useful for determining planes to investigate molecules that are unique to that strain, it still requires manual inspection of a loadings plot to investigate those masses. Automating this procedure would drastically simplify the strain prioritization by metabolomics. As an example, in Figure 3, the Scores and loadings plots are shown for PC13 vs. PC15, and clearly show that WMMB-499 separates from the other strains in the scores plot, while compounds unique to that strain are easily identified in the loadings plot.

For true statistical analyses, replicates would be used, as was done for Figure 1, but to use PCA as a survey tool replicates are not necessary. In part, we envision the use of this approach on environmental collections where replicates are included as a result of the cultivation process (i.e., environmental replicates). Additionally, we found that introducing replicates into PCA can result in large datasets that are computationally challenging to generate PCA models. However, averaging replicates after feature finding provides an excellent alternative. All bacteria in this study were cultivated from marine sponges and ascidians. Table S1 in the supplementary material provides details about the origins of each strain. We used two major datasets to demonstrate aspects about the identification of unique molecular features. Dataset one comprises 36 bacterial strains that include 32 *Streptomyces* spp., two *Nocardia* spp., one *Actinomadura* sp. (WMMB-499), and one *Nocardiopsis* sp. Dataset two comprises 19 bacterial strains, including 18 *Streptomyces* spp. and one *Actinomadura* sp. (WMMB-499), and was used to illustrate the impact of the group used for PCA.

**Figure 4:**
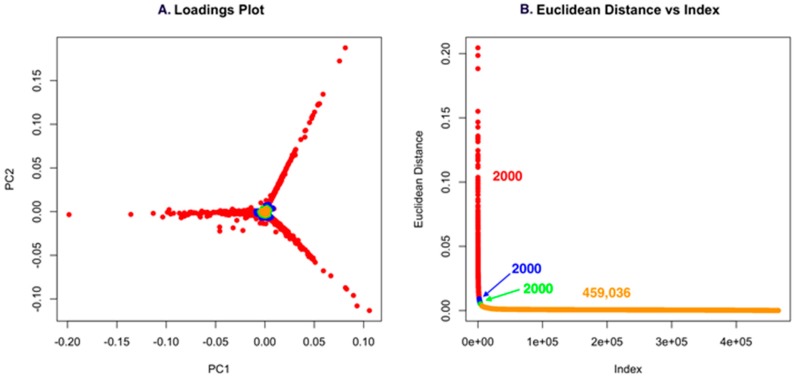
(**A**) The loadings plot for Dataset one with the 2000 most unique features colored in red; (**B**) The Euclidean distance vs. index plot is color-coded to match the points in the loadings plot. This example shows that 459,036 features are located in the center of the PCA plot and, based on Euclidean distance, contribute little to variance, meaning that they are not unique to any strain.

**Figure 5:**
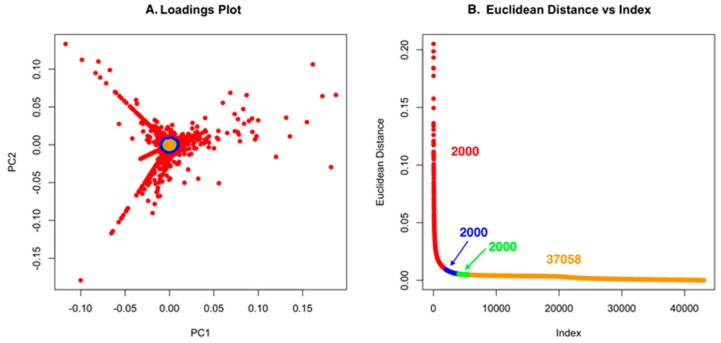
(**A**) The loadings plot for Dataset two with the 2000 most unique features colored in red; (**B**) The Euclidean distance vs. index plot is color-coded to match the points in the loadings plot. This example shows that 37,058 features are located in the center of the PCA plot and, based on Euclidean distance, contribute little to variance, meaning that they are not unique to any strain.

Using low S/N or ion intensity thresholds offers the best route for detecting low intensity masses that could be important for discovery. However, this can also introduce a significant number of features that are not relevant, but the strength of PCA is that “background” ions that are present across strains have little to no impact on the ability to detect unique molecules. For Dataset one, using a low S/N threshold, we identified 465,036 features (Figure 4). While this is an unusually high number of data points, it makes an important point about the use of PCA in that it allows low S/N thresholds to be used to avoid missing a compound. However, if minor features are common across the strains analyzed, they will not contribute to variance and will have a small Euclidean distance. In Figure 4A and Figure 5A, the scores plot is color-coded to indicate the relationship with panel B, which shows the Euclidean distance. Essentially, one can describe the significant data using about 2000 data points. The goal of PoPCAR as described in this manuscript is to facilitate identifying those significant data points in the background of a large number of features.

### 2.2. Dependence of the Analysis on the Group

**Figure 6:**
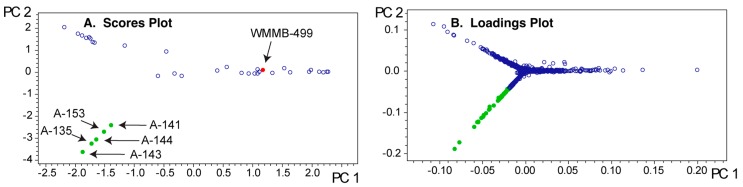
PCA model for Dataset one. (**A**) In contrast to Figure 3A, the scores plot showing PC1 vs. PC2 gives the illusion that WMMB-499 is not particularly unique; (**B**) The unique molecular features that can be identified from PC1–PC2 are associated with a group of similar strains highlighted in the lower left of the scores plot.

**Figure 7:**
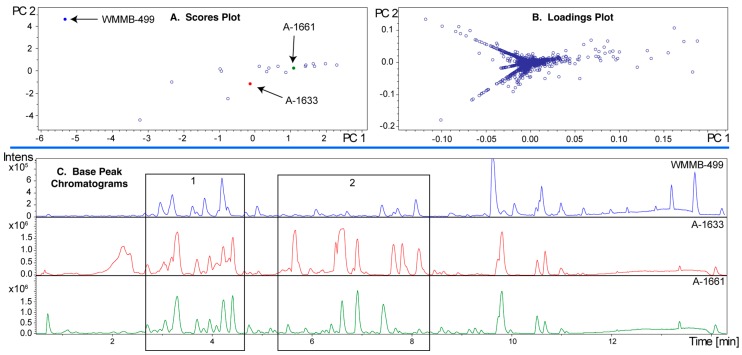
PCA model for Dataset two. (**A**) In contrast to Figure 6A, the scores plot showing PC1 vs. PC2 shows that WMMB-499 is unique relative to the other strains; (**B**) Similarly, the loadings plot provides clear evidence for unique molecular features associated with WMMB-499. (**C**) Examples of base peak chromatograms showing that WMMB-499 yielded lower overall ion intensity.

Since PCA provides a model of the covariance among the LC-MS data, that covariance and the PCA model will depend on the collection of strains used for the analysis. For strain prioritization, we tend to group about 40–50 strains with similar phenotypic features for each PCA analysis. Based on our experience, a good rule of thumb is to have ~50 strains because the PCA can become uninformative beyond this limit. To illustrate the group effect, we analyzed WMMB-499 in two different groups that were comprised mostly of *Streptomyces* spp. (Figure 6 and Figure 7). Each of the two PCA models show markedly different outcomes in PC1–PC2 for WMMB-499. Figure 3 and Figure 6 represent the same PCA model. As highlighted in Figure 3A, a higher set of planes was required to separate strain WMMB-499 from the remaining strains. In Figure 6A (Dataset one), the scores plot for PC1–PC2 indicates that WMMB-499 is not unique, and similarly unique molecular features associated with WMMB-499 cannot be easily identified. Alternatively, Figure 7 shows a different PCA model for Dataset two with fewer strains (19 vs. 36), in which WMMB-499 is clearly unique, and unique molecular features can easily be identified in the loadings plot. These examples highlight the complexities of identifying unique molecular features using PCA. On the other hand, using the PoPCAR script, the list of masses that are unique for WMMB-499 is the same for both datasets (Table S3).

In Figure 7C, base peak chromatograms are shown for three strains in the analysis. Importantly, the ion intensity for WMMB-499 is the lowest among the three. Without some level of scaling to mitigate the impact of ion intensity, WMMB-499 would have fewer unique features. While PoPCAR can significantly reduce the effort, and maximize utilization of information within PCA models, the outcome will depend on generating peak lists.

Overall, Figure 6 and Figure 7 highlight that the discovery of interesting molecules from a collection of bacterial strains is dependent on the strains analyzed. For our workflow, we analyze *Streptomyces* and other actinomycetes that grow well and sporulate on solid agar-based media together due to identical processing methods. Additionally, we analyze *Micromonospora*-like strains together as they require liquid media in the presence of both iron and a resin such as HP20 or XAD for productivity (see Section 4 for more details). Mounting evidence both from our work and genomics suggest that identifying unique molecules within the context of a collection of similar strains is highly advantageous for discovery, but simple ways to do this have been lacking. Hence, we have generated the PoPCAR script to streamline this process.

### 2.3. PoPCAR

**Figure 8:**
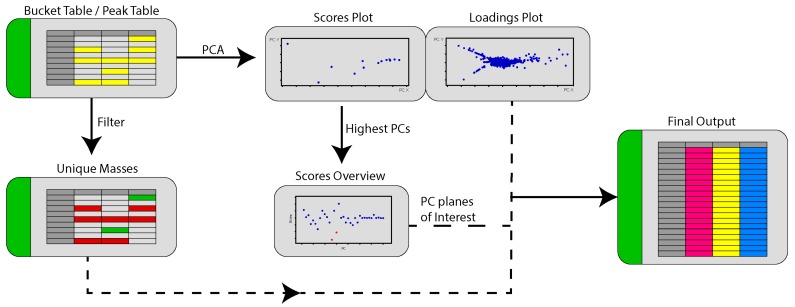
An overview of the process used by PoPCAR to generate a ranked list of unique features found for a particular strain in a PCA model. The final output is in Excel format and can be easily viewed.

Given the complexities of manually identifying planes where unique masses for each strain from a particular analysis could be found, we sought to automate the workflow using a script written in R and named it PoPCAR for Planes of Principal Component Analysis in R. The general flow of the script can be found in Figure 8. The underlying idea behind PoPCAR is that masses unique to each strain can be identified simply by locating those in a bucket table (i.e., peak table or peak list), which can be generated using vendor-supplied software such as Bruker ProfileAnalysis or open source tools such as MZmine 2 or XCMS. However, the peak tables are often too large to manually view and identify unique features. On the other hand, PCA can simplify this process because it groups features that are identical across strains into the center of the plot, meaning that those features do not contribute to the variance within the model. Therefore, by first using PCA and then filtering the peak table for unique masses, a streamlined output can be generated that highlights masses that are unique and identifies the plane where those masses can be identified. Essentially, there are as many PCs as there are strains in the dataset. Each strain “stands out” in a certain PC plane on the scores plot and the corresponding loadings plot shows the metabolites that contribute to that strain standing out in that PC plane. The script automates the process of finding this plane for each strain and identifying the unique metabolites produced by that strain. As an example, there are 36 strains in the dataset used in Figure 6 and so there are 36 possible PCs. The script would analyze and pull out the unique metabolites associated with each strain in the dataset.

What is particularly useful about this approach is that it greatly facilitates evaluating the effect of peak table generation parameters and PCA parameters on the identity of unique masses. In our example, we used Dataset one as an example where the S/N threshold was too low and the peak list was unusually large. Having some knowledge about the expected number of masses and the use of PoPCAR would signal that a lower S/N threshold or intensity threshold should be used for generating the peak table and the PCA. With respect to PCA, PoPCAR provides a means to compare the outcome of different PCA models. Normally, it is quite challenging or impossible to compare the outcomes of PCA under different conditions, especially for big datasets. Finally, we provide an example where PoPCAR can be integrated with the Antibase database to perform fairly automated dereplication and identify peaks that do not match masses in Antibase, signaling a potential novel molecule.

## 3. Discussion

Redirecting efforts from dereplication toward discovery-focused strategies has the potential to increase the discovery rate of new and novel natural products. We have mounting evidence that discovery-based strategies—such as the one discussed in this paper—increase the discovery rate, and make sense based on what has been shown about the distribution of GCFs. The simple script that we developed will assist researchers by greatly reducing the total number of mass features that need to be considered. Currently, there are no simple ways of analyzing a group of bacterial strains to retrieve masses that are only unique to each strain, at least for large datasets. For actinomycetes, we can use the idea that common metabolites are likely not novel to leverage PCA for an internal pseudo-dereplication process in an untargeted fashion.

As an example of the output of the script, we compare the top 60 unique metabolites for WMMB-499 from the two datasets (see Table S3 in Supplementary Materials). The table illustrates a couple of important points. Within the small dataset, we identified more unique molecular features. This is expected because, in general, the more strains analyzed, the fewer unique features appear. This occurs because in very large datasets, intergroup and intragroup variances tend to become similar. In other words, the model is unable to highlight the differences between strains, so nothing stands out as a unique feature. The other important point illustrated by the table is that the most unique feature corresponds to *m*/*z* 1185.7445, at a retention time of 9.65 min, and is a putative novel peptide. While we isolated the peptide, we were unable to find a suitable solvent for NMR studies. In other words, the peptide seems conformationally flexible and yields broad signals in the NMR. Another interesting feature that stands out in both datasets is the fragment ion that corresponds to Forazoline A at *m*/*z* 760.2949. While the major ion is due to the loss of a forosamine sugar, the fragment is still an indicator of a novel molecule. In general, if the skeleton is novel, even fragments will be unusual due to an unusual molecular formula. Upon closer inspection, an [M + H]^+^ of 901.4205 could be identified.

In summary, we provide a script, PoPCAR that can greatly assist with analyses of PCA models that are generated predominantly from secondary metabolites, where little is known about the molecules, their concentration, or their ionization efficiency. In theory, the script could also be used to generate unique masses across larger collections by generating lists from multiple PCA models. Since the prioritization of bacterial strains prior to whole genome sequencing and/or screening is typically performed, we anticipate that PoPCAR will be useful for that purpose. The script can also be used with minor modifications on other kinds of data such as human, plant, or fungal metabolites, or even with NMR metabolomics.

## 4. Materials and Methods

### 4.1. Bacterial Cultivation

Sponge and Ascidian specimens were collected from the Florida Keys. For cultivation, a sample of each host (1 cm^3^) was rinsed with sterile seawater, macerated using a sterile pestle in a micro-centrifuge tube, and dilutions were made in sterile seawater, with vortexing between steps to separate the bacteria from heavier tissues. Dilutions were separately plated on seven distinct media: R2A [32], R2A+Desferrioxamine, Bonito, ISP3 [33], M4 [34], Gauze 1 [35], ISP2 [33] (see recipes in Table S2 in supplementary material), supplemented with artificial sea water [36] and M4 [37]. Media were supplemented with 50 μg/mL cycloheximide and 25 μg/mL nalidixic acid. Plates were incubated at 28 °C for at least 28 days.

For marine members of *Micromonosporaceae*, we found that these strains produce on the order of 500 mg to 1 g per liter of iron chelators, which mask other compounds in the LC-MS analyses. The production of these can be suppressed via the addition of iron. HP20 was also added in liquid media for members of this family for optimum productivity.

### 4.2. Agar-based Media for LC-MS Profiling

Each strain was inoculated onto ISP2 media supplemented with artificial sea water in Petri dishes and incubated at 28 °C for 10 days or until sporulation was observed.

### 4.3. Sample Preparation for UHPLC/HRESI-TOF-MS

Two cores (8 mm in diameter) of bacteria and agar were obtained from each plate, placed directly into MeOH (2 mL), and extracted for 30 min. The extract was transferred into a clean glass vial and evaporated using a SpeedVac concentrator; the extract was dissolved in MeOH (100 μL) and diluted with H_2_O (1 mL). The solution was then placed on a Gilson GX-271 liquid handling system for automated SPE (Biotage: EVOLUTE ABN, 25 mg absorbent mass, 1 mL reservoir volume), washed using H_2_O (1 mL) to remove media components, and eluted with MeOH (1 mL) directly into an LC-MS-certified vial.

### 4.4. UHPLC/HRMS Analysis.

LC-MS data were acquired using a Bruker maXis 4G ESI-Q-TOF mass spectrometer coupled with a Waters Acquity UPLC system. A gradient of MeOH and H_2_O (containing 0.1% formic acid) was employed with a flow rate of 0.3 mL/min on an RP C-18 column (Phenomenex Kinetex 2.6 μm, 2.1 mm × 100 mm). The gradient started from MeOH/H_2_O (10%/90%), followed by a linear gradient to reach MeOH/ H_2_O (97%/3%) in 12 min, and was held for 2 min at MeOH/H_2_O (97%/3%). Full scan mass spectra (*m*/*z* 150–1550) were measured in positive ESI mode [38]. The mass spectrometer was operated using the following parameters: capillary, 4.5 kV; nebulizer pressure, 4.0 bar; dry gas flow, 6.0 L/min; dry gas temperature, 200 °C; scan rate, 2 Hz. Tune mix (Agilent, Santa Clara, CA, USA, ESI-L low concentration) was introduced through a divert valve at the end of each chromatographic run. The spectra acquired on tune mix were used for mass calibration of the data prior to PCA.

### 4.5. Data Processing and PCA

PCA was performed using Bruker Compass ProfileAnalysis 2.3 (Billerica, MA, USA). Find Molecular Features was applied to LC-MS data under these parameters: S/N threshold, 5; correlation coefficient threshold, 0.7; minimum compound length, 10; smoothing width, 1. The LC-MS datasets were evaluated in a time range from 2 to 14 min and in a mass range from *m*/*z* 150 to 1500. Advanced bucketing was employed using ΔRT = 0.33 min and Δ*m*/*z* = 4 ppm as parameters. Sum of bucket values was used for normalization, and Pareto scaling [39] was applied.

### 4.6. PoPCAR

RStudio version 1.0.136 [40] with R version 3.3.3 (6 March 2017)—“Another Canoe” [41]—was used for developing the script. In brief, the bucket table from ProfileAnalysis was exported as a tab delimited *.txt file. It was then imported into R using *data.table* [42]. The row and column names were cleaned up to improve readability using *stringr* [43] package. PCA was then performed on this data table followed by different manipulations as described in the text. An excel workbook was written to the disk using the *xlsx* [44] package. The algorithm and additional information may be found in the Supplementary Material.

## Supplementary Material

The following are available online at www.mdpi.com/2218-1989/7/3/34/s1, Table S1: Spreadsheet with details of strains used, Table S2: Media recipes, Table S3: Unique metabolites of WMMB-499 from two different datasets, S4: PoPCAR script.
