# Supplementary material for: Natural Product Discovery Using Planes of Principal Component Analysis in R (PoPCAR)
Source: Metabolites. 2017 Jul 13;7(3):34. doi: 10.3390/metabo7030034 (PMC5618319; doi:10.3390/metabo7030034)
Supplement: Supplementary file 1 [file metabolites-07-00034-s001.pdf]

metabolites

MDPI

Supplementary Material: PoPCAR – Planes of Principal Component Analysis in R

Shaurya Chanana, Chris S. Thomas, Doug R. Braun, Yanpeng Hou, Thomas P Wyche, and Tim S Bugni

Table S1: Excel spreadsheet with details of strains used

Table S2: Media recipes

Table S3: Unique metabolites of WMMB-499 from two different datasets

S4: PoPCAR script

Table S1 Spreadsheet with details of strains used

Dataset 1: 36 Strains

Strain		Genus	Sponge	Media	Location	Date Collected
A	133	*Nocardia levis strain 13679H*	*Spheciospongia vesparium*	R2A+DesI	24° 41.638', 81° 26.614'	8/16/11
A	135	*Streptomyces*	*Spheciospongia vesparium*	R2A	24° 41.638', 81° 26.614'	8/16/11
A	139	*Streptomyces*	*Ircinia campara*	R2A	24° 38.977', 81° 25.421'	8/16/11
A	140	*Streptomyces*	*Ircinia campara*	R2A	24° 38.977', 81° 25.421'	8/16/11
A	141	*Streptomyces*	*Ircinia campara*	R2A+DesI	24° 38.977', 81° 25.421'	8/16/11
A	142	*Streptomyces*	*Spongia pertusa*	R2A	24° 38.927', 81° 25.421'	8/16/11
A	143	*Streptomyces*	*Ircinia campara*	R2A	24° 38.977', 81° 25.421'	8/16/11
A	144	*Streptomyces*	*Ircinia ramosa*	R2A	24° 38.927', 81° 25.421'	8/16/11
A	149	*Streptomyces*	*Ircinia ramosa*	R2A+DesI	24° 38.927', 81° 25.421'	8/16/11
A	150	*Streptomyces*	*Ircinia ramosa*	R2A+DesI	24° 38.927', 81° 25.421'	8/16/11
A	153	*Streptomyces*	*Tedonia ignis*	M4	24° 33.416', 81° 21.611'	8/16/11
A	154	*Streptomyces*	*Tedonia ignis*	M4	24° 33.416', 81° 21.611'	8/16/11
A	156	*Streptomyces*	*Tedonia ignis*	R2A	24° 33.416', 81° 21.611'	8/16/11
A	157	*Streptomyces*	*Tedonia ignis*	R2A+DesI	24° 33.416', 81° 21.611'	8/16/11
A	160	*Streptomyces*	*Tedonia ignis*	R2A+DesI	24° 33.416', 81° 21.611'	8/16/11
A	162	*Nocardia neocaledoniensis strain DSM 44717*	*Tedonia ignis*	M4	24° 33.416', 81° 21.611'	8/16/11
A	165	*Streptomyces sp. CNQ-301_SD01*	*Chondrilla nucula*	R2A+DesI	24° 39' 18", 81° 17' 51"	2/10/10
B	497	*Streptomyces sp. HY26(2010)*	*Ecteinascidia turbinata*	M4	24° 39.439', 81° 26.217'	8/15/11
B	499	*Actinomadura sp. 13679C*	*Ecteinascidia turbinata*	Isp2	24° 37.487', 81° 27.443'	10/11/10
B	500	*Streptomyces*	*Ecteinascidia turbinata*	Isp2	24° 37.487', 81° 27.443'	10/11/10
B	503	*Nocardiopsis sp. JAJ60*	*Ecteinascidia turbinata*	M4	24° 37.487', 81° 27.443'	10/11/10
B	507	*Streptomyces*	*Ecteinascidia turbinata*	R2ADesI	24° 39.439', 81° 26.217'	8/15/11
B	510	*Streptomyces sp. SCSIO 1666*	*Ecteinascidia turbinata*	Isp2	24° 39.439', 81° 26.217'	8/15/11
B	514	*Streptomyces chryseus strain HBUM174847*	*Didemnum psammathode*	Isp2	24° 41.372', 81° 26.797'	8/15/11
B	515	*Streptomyces*	*Didemnum psammathode*	R2A	24° 41.372', 81° 26.797'	8/15/11
B	518	*Streptomyces sp. CNQ-147_SD01*	*Ecteinascidia turbinata*	M4	24° 39.439', 81° 26.217'	8/15/11
B	519	*Streptomyces*	*Ecteinascidia turbinata*	M4	24° 39.439', 81° 26.217'	8/15/11
B	520	*Streptomyces*	*Ecteinascidia turbinata*	M4	24° 39.439', 81° 26.217'	8/15/11
B	521	*Streptomyces sp. G2X8*	*Ecteinascidia turbinata*	M4	24° 39.439', 81° 26.217'	8/15/11

B	522	*Streptomyces*	*Ecteinascidia turbinata*	M4	24° 39.439', 81° 26.217'	8/15/11
B	523	*Streptomyces*	*Ecteinascidia turbinata*	M4	24° 39.439', 81° 26.217'	8/15/11
B	524	*Streptomyces*	*Ecteinascidia turbinata*	Isp2	24° 39.439', 81° 26.217'	8/15/11
B	526	*Streptomyces sp. 172205*	*Ecteinascidia turbinata*	R2A	24° 39.439', 81° 26.217'	8/15/11
B	528	*Streptomyces*	*Ecteinascidia turbinata*	Isp2	24° 39.439', 81° 26.217'	8/15/11
B	530	*Streptomyces*	*Ecteinascidia turbinata*	Isp2	24° 39.439', 81° 26.217'	8/15/11
B	531	*Streptomyces sp. 172205*	*Ecteinascidia turbinata*	Isp2	24° 39.439', 81° 26.217'	8/15/11

Dataset 2**:** 12 Strains

Strain		Genus	Sponge	Media	Location	Date collected
A	1578	*Streptomyces*	*Suberites sp.*	R2A	27° 28' 45.7", 80° 18' 42.8"	8/7/13
A	1580	*Streptomyces*	*Suberites sp.*	Isp3	27° 28' 45.7", 80° 18' 42.8"	8/7/13
A	1585	*Streptomyces*	*Suberites sp.*	Gauze 1	27° 28' 45.7", 80° 18' 42.8"	8/7/13
A	1589	*Streptomyces*	*Suberites sp.*	Bonito	27° 28' 45.7", 80° 18' 42.8"	8/7/13
A	1595	*Streptomyces*	*Suberites sp.*	Isp2	27° 28' 45.7", 80° 18' 42.8"	8/7/13
A	1606	*Streptomyces*	*Suberites sp.*	Isp3	27° 28' 45.7", 80° 18' 42.8"	8/7/13
A	1608	*Streptomyces*	*Suberites sp.*	Gauze 1	27° 28' 45.7", 80° 18' 42.8"	8/7/13
A	1609	*Streptomyces*	*Suberites sp.*	Gauze 1	27° 28' 45.7", 80° 18' 42.8"	8/7/13
A	1611	*Streptomyces*	*Suberites sp.*	Bonito	27° 28' 45.7", 80° 18' 42.8"	8/7/13
A	1612	*Streptomyces*	*Suberites sp.*	Gauze 1	27° 28' 45.7", 80° 18' 42.8"	8/7/13
A	1613	*Streptomyces sp. NA684*	*Suberites sp.*	Gauze 1	27° 28' 45.7", 80° 18' 42.8"	8/7/13
A	1633	*Streptomyces sp. QZGY-A33*	*Haliclona sp.*	R2A	27° 28' 45.7", 80° 18' 42.8"	8/7/13
A	165	*Streptomyces sp. CNQ-301_SD01*	*Chondrilla nucula*	R2A+DesI	24° 39' 17.90", 81° 17' 51.09"	2/10/10
A	1657	*Streptomyces*	*Haliclona sp.*	Isp3	27° 28' 45.7", 80° 18' 42.8"	8/7/13
A	1661	*Streptomyces*	*Lissodendoryx sigmata*	HV Gent	27° 28' 45.7", 80° 18' 42.8"	8/7/13
A	1666	*Streptomyces*	*Haliclona sp.*	R2A	27° 28' 45.7", 80° 18' 42.8"	8/7/13
A	2171	*Streptomyces*	*Cinachyra alloclada*	Isp 2	24° 33.035", 81° 21.872"	8/12/14
B	499	*Actinomadura sp. 13679C*	*Ecteinascidia turbinata*	Isp2	24° 37.487', 81° 27.443'	10/11/10
B	705	*Streptomyces sp. FXJ6.141*	*Eudistoma olivaceum*	Isp2	24° 33.416', 81° 21.611'	9/1/11

Table S2 Media Recipes

Media	Recipe (all ingredients in g/L unless specified otherwise)
ASW-A	Soluble starch – 20<br>Glucose – 10<br>Peptone – 5<br>Yeast extract – 5<br>Calcium carbonate ($CaCO_3$) – 5<br>Artificial seawater – 1L
ISP2	Yeast extract – 4<br>Malt extract – 10<br>Dextrose – 4<br>Agar – 15<br>Artificial seawater – 1L
ISP3	Oatmeal – 20<br>Agar – 15<br>Artificial seawater – 1L
R2A	Yeast extract – 0.5<br>Peptone – 0.5<br>Casamino acids – 0.5<br>Dextrose – 0.5<br>Soluble Starch – 0.5<br>Sodium Pyruvate – 0.3<br>Dipotassium Phosphate – 0.3<br>Magnesium Sulphate – 0.05<br>Agar – 15<br>Artificial seawater – 1L
R2A+DesI	R2A medium with desferrioxamine added at 0.1mg/ml and Ferrous Sulfate ($FeSO_4$) at equimolar ratio (Desferrioxamine was purified from laboratory grown micromonospora)
Gauze1	Starch – 20<br>Potassium Nitrate (KNO_3) – 1<br>Dihydrogen Phosphate (H_2PO_4) – 0.5<br>Ferrous Sulfate Heptahydrate ($FeSO_4.7H_2O$) – 0.01<br>Agar – 15<br>Artificial seawater – 1L
HV	Humic Acid (dissolved in 10 mL 0.2N NaOH) – 1<br>Sodium Phosphate dibasic (Na_2HPO_4) – 0.5<br>Potassium Chloride (KCl) – 1.71<br>Magnesium Sulfate Heptahydrate ($MgSO_4.7H_2O$) – 0.05<br>Ferrous Sulfate Heptahydrate ($FeSO_4.7H_2O$) – 0.01<br>Calcium Carbonate (CaCO3) – 0.02<br>Yeast extract – 0.5<br>Agar – 15<br>Artificial seawater – 1L

M4	L-asparagine – 0.1<br>Potassium Phosphate Dibasic (K_2HPO_4) – 0.5<br>Ferrous Sulfate ($FeSO_4$) – 0.001<br>Magnesium Sulfate ($MgSO_4$) – 0.1<br>Peptone – 2<br>Sodium Propionate – 4<br>Sodium Chloride (NaCl) – 20<br>Agar - 15
Bonito	Ground Bonito flakes - 2<br>Glucose – 2<br>Peptone – 2<br>Monopotassium Phosphate (KH_2PO_4) – 1<br>Ammonium Chloride (NH_4Cl) – 1<br>Agar – 15<br>Artificial seawater – 1L

Table S3: Unique metabolites of WMMB-499 from two different datasets

Dataset one

	rt	m	Intensity	PC13	PC15	Euclidean Distance
1	9.65	1185.7445	2698359.736	0.136	0.096	0.166
6	8.10	366.1387	742020.680	0.071	0.050	0.087
8	2.97	932.4149	624261.558	0.066	0.046	0.080
13	7.74	366.1386	370803.017	0.050	0.035	0.062
18	13.00	753.3849	321971.682	0.047	0.033	0.058
20	9.87	596.3688	267946.921	0.043	0.030	0.052
22	6.72	641.3327	233635.064	0.040	0.028	0.049
23	3.91	900.4246	228597.130	0.040	0.028	0.048
24	9.85	1199.7587	228137.565	0.040	0.028	0.048
26	8.10	360.3120	213832.320	0.038	0.027	0.047
#N/A	7.66	587.2582	203614.781	0.037	0.026	0.046
28	9.87	1192.7331	201358.423	0.037	0.026	0.045
32	2.66	185.0926	158470.329	0.033	0.023	0.040
41	6.21	929.4371	132845.281	0.030	0.021	0.037
45	3.13	932.4142	117565.620	0.028	0.020	0.035
47	13.30	731.4029	113453.726	0.028	0.020	0.034
49	5.69	1252.6106	104991.517	0.027	0.019	0.033
52	10.92	565.3744	102434.541	0.027	0.019	0.032
55	6.45	921.3986	95921.162	0.026	0.018	0.031
57	5.01	841.4043	94708.326	0.026	0.018	0.031
58	6.44	323.2230	91902.563	0.025	0.018	0.031
62	13.96	313.3117	87590.257	0.025	0.017	0.030
64	13.10	731.4026	86211.563	0.024	0.017	0.030
65	2.97	970.3611	85396.095	0.024	0.017	0.030
69	10.39	611.3793	82863.307	0.024	0.017	0.029
70	9.87	1169.7477	80655.323	0.024	0.017	0.029
72	7.60	375.2182	78305.669	0.023	0.016	0.028
73	5.77	556.2426	77476.380	0.023	0.016	0.028
76	9.64	1275.7127	72058.354	0.022	0.016	0.027
77	6.55	325.2389	71985.792	0.022	0.016	0.027
79	7.97	573.2784	69611.950	0.022	0.015	0.027
80	7.41	333.2069	69439.181	0.022	0.015	0.027

81	5.30	1135.5501	69128.198	0.022	0.015	0.027
82	8.19	418.3539	67130.992	0.021	0.015	0.026
85	2.97	976.3776	64446.167	0.021	0.015	0.026
89	2.66	167.0818	61616.216	0.021	0.014	0.025
91	7.60	339.1966	60876.766	0.020	0.014	0.025
92	8.15	365.2337	60489.765	0.020	0.014	0.025
93	8.97	684.2932	60275.531	0.020	0.014	0.025
94	4.39	967.4770	60123.495	0.020	0.014	0.025
95	5.28	881.4235	59802.145	0.020	0.014	0.025
96	7.59	997.3969	58845.007	0.020	0.014	0.025
97	6.04	396.2759	58219.584	0.020	0.014	0.024
98	5.18	319.1918	56916.909	0.020	0.014	0.024
100	6.41	899.3931	55203.044	0.019	0.014	0.024
101	7.42	315.1968	54764.211	0.019	0.014	0.024
104	3.78	946.4305	54581.076	0.019	0.014	0.024
105	10.08	1153.7523	54470.504	0.019	0.014	0.024
109	8.84	383.2444	52936.319	0.019	0.013	0.023
110	7.38	317.1761	52483.665	0.019	0.013	0.023
111	5.27	917.4031	52438.745	0.019	0.013	0.023
112	9.65	1229.7071	52404.191	0.019	0.013	0.023
116	9.25	1209.7035	50863.095	0.019	0.013	0.023
117	5.06	1080.5622	50282.592	0.019	0.013	0.023
119	7.05	760.2921	49588.062	0.018	0.013	0.023
121	10.45	1161.7292	48637.834	0.018	0.013	0.022
126	11.41	525.3813	46543.878	0.018	0.013	0.022
127	10.57	581.3690	46084.313	0.018	0.013	0.022
2355	7.25	353.2338	45783.696	0.018	0.012	0.022
134	5.27	980.3331	45058.068	0.018	0.012	0.022

Dataset two

	rt	m	Intensity	PC1	PC2	Euclidean Distance
1	9.65	1185.7445	4728561.654	-0.117	0.133	0.177
#N/A	13.70	536.1693	3355964.385	-0.099	0.112	0.149
#N/A	13.24	445.1227	2390787.202	-0.083	0.095	0.126
#N/A	13.70	519.1425	2105565.894	-0.078	0.089	0.118
#N/A	4.24	514.2323	1760065.668	-0.071	0.081	0.108
2	8.10	366.1387	1300304.954	-0.061	0.070	0.093
#N/A	10.59	313.1450	1247219.543	-0.060	0.068	0.091
3	2.97	932.4149	1093945.788	-0.056	0.064	0.085
#N/A	10.08	315.0942	1013291.325	-0.054	0.062	0.082
#N/A	7.42	351.2183	893387.445	-0.051	0.058	0.077
#N/A	12.92	393.2994	811122.315	-0.048	0.055	0.073
#N/A	11.47	261.2437	682117.560	-0.044	0.051	0.067
4	7.74	366.1386	649789.169	-0.043	0.049	0.066
#N/A	13.20	367.3019	606507.029	-0.042	0.048	0.064
#N/A	6.10	857.3993	570593.994	-0.041	0.046	0.062
#N/A	12.91	391.2864	564986.935	-0.040	0.046	0.061
#N/A	12.02	315.2547	564762.895	-0.040	0.046	0.061
5	13.00	753.3849	564217.932	-0.040	0.046	0.061
#N/A	11.06	287.2232	492973.157	-0.038	0.043	0.057
6	9.87	596.3688	469545.820	-0.037	0.042	0.056

#N/A	12.85	353.2861	445228.379	-0.036	0.041	0.054
7	6.72	641.3327	409418.282	-0.034	0.039	0.052
8	3.91	900.4246	400589.888	-0.034	0.039	0.052
9	9.85	1199.7587	399784.554	-0.034	0.039	0.052
#N/A	10.97	389.1136	392336.732	-0.034	0.038	0.051
10	8.10	360.3120	374716.275	-0.033	0.038	0.050
#N/A	13.24	462.1489	367771.030	-0.033	0.037	0.049
12	9.87	1192.7331	352857.220	-0.032	0.036	0.048
#N/A	12.08	329.2706	313268.716	-0.030	0.034	0.046
#N/A	12.46	379.2837	285082.040	-0.029	0.033	0.044
#N/A	13.70	550.1842	282726.590	-0.029	0.033	0.043
13	2.66	185.0926	277700.824	-0.028	0.032	0.043
#N/A	8.80	350.9882	273668.101	-0.028	0.032	0.043
#N/A	4.68	217.0989	269653.543	-0.028	0.032	0.042
#N/A	4.31	299.0861	251918.039	-0.027	0.031	0.041
#N/A	3.21	453.2727	250979.492	-0.027	0.031	0.041
#N/A	9.49	1183.7269	242823.219	-0.027	0.030	0.040
#N/A	11.57	321.2433	235768.981	-0.026	0.030	0.040
#N/A	12.69	279.2311	233922.163	-0.026	0.030	0.039
#N/A	11.49	305.2701	233873.722	-0.026	0.030	0.039
14	6.21	929.4371	232795.907	-0.026	0.030	0.039
#N/A	13.11	307.2623	230731.104	-0.026	0.029	0.039
#N/A	10.83	399.2528	217767.050	-0.025	0.029	0.038
#N/A	6.60	933.3983	210416.110	-0.025	0.028	0.037
15	3.13	932.4142	206020.079	-0.024	0.028	0.037
#N/A	4.35	176.0710	201079.084	-0.024	0.027	0.037
16	13.30	731.4029	198814.462	-0.024	0.027	0.036
#N/A	11.37	311.2208	197264.346	-0.024	0.027	0.036
17	5.69	1252.6106	183985.425	-0.023	0.026	0.035
#N/A	9.65	1250.7310	183488.903	-0.023	0.026	0.035
#N/A	5.45	899.3920	182635.128	-0.023	0.026	0.035
18	10.92	565.3744	179504.621	-0.023	0.026	0.035
#N/A	3.86	543.2235	176362.004	-0.023	0.026	0.034
#N/A	10.08	223.0642	174279.035	-0.022	0.026	0.034
19	6.45	921.3986	168090.682	-0.022	0.025	0.033
#N/A	5.43	760.2925	166358.912	-0.022	0.025	0.033
20	5.01	841.4043	165965.328	-0.022	0.025	0.033
21	6.44	323.2230	161048.555	-0.022	0.025	0.033
#N/A	13.70	557.0980	159661.928	-0.022	0.025	0.033
#N/A	9.94	452.8719	159032.193	-0.021	0.024	0.033

Note: the leftmost column indicates which row of the other table that 'm' can be found in. For example, from the first table, the second mass – 366.1387 *m*/*z* – can be found in the sixth row of the second table. The reverse is indicated in the second table referencing the first table. The green cell color indicates matches, while '#N/A' indicates that it is not present in the other table. Two dark green rows have been highlighted indicating the masses highlighted in the main text.

S4: PoPCAR Script

Shaurya Chanana

4/29/2017

Libraries

```
library(data.table)
library(stringr)
library(xlsx)
```

Definitions

Note: here, use bucket table according to dataset being analyzed.

```
setwd("~/Lab/Bucket Tables/")
# bucket_table_name = "A133-B531.txt"
# bucket_table_name = "smaller-dataset-bucket.txt"
FLAG = 0 # Set to 1 if you want to include Antibase
if (FLAG == 1) {
    ppm = 4 # Can be changed to allow user to input desired accuracy
    PROTON = 1.6726231 / 1.6605402 # mass in kg to mass in u
    ELECTRON = (9.1093897 / 1.6605402) / 10000
    SODIUM = 22.989768
    SODIUM_Plus = SODIUM - ELECTRON
}
```

Functions

General Functions

Sorts the row and returns it with the original indices

```
fd.sort <- function(x) {
    sorted = sort(x, decreasing = T, index.return = T)
    return(sorted)
}
```

Pareto scales the data:
1. Subtract the column mean from each element of the column.
2. Divide each element of the column by the square root of the standard deviation of that column.

```
ParetoScale <- function(x) {
    return(apply(
        X = x,
        MARGIN = 2,
        FUN = function(x)
            (x - mean(x)) / sqrt(sd(x))
    ))
}
```

Takes a two-column data frame or matrix and adds a third column called 'Euclidean Distance' into it.

```
add_euclidean_distance <- function(x) {
    x = cbind(x, sqrt((x[, 1]) ^ 2 + (x[, 2]) ^ 2))
    colnames(x)[3] = "Euclidean Distance"
    return(x)
}
```

Returns a row without the zero elements

```
filter_zero <- function(x) {
    x = x[which(x != 0)]
    return(x)
}
```

Lookup Functions

Takes an input of a vector of length = 3 and sees if both indices refer to the same (physical) strain in the bucket table. If true it returns the the first of the two row indices it received otherwise it returns -1.

```
lookup_function_triplicate <- function(x) {
    a = rownames(f.d)[x[1]]
    b = rownames(f.d)[x[2]]
    c = rownames(f.d)[x[3]]
    if (a == b & b == c) {
        result = x[1]
    }
    else {
        result = -1
    }
    return(result)
}
```

Same as the triplicate version except takes input of length = 2.

```
lookup_function_duplicate <- function(x) {
    c = rownames(f.d)[x[1]]
    d = rownames(f.d)[x[2]]
    if (c == d) {
        result = x[1]
    }
    else {
        result = -1
    }
    return(result)
}
```

Function to figure out which columns have unique chemistry (unique masses. It takes an input of a column from the bucket table and outputs the row name for that column that has a unique mass in it using lookup functions defined for duplicate and triplicate data.

```
different_function <- function(x) {
    # if there are non-zero elements, this returns the indices of those
```

```
elements in the list
    n = which(x != 0)
    # if there is only one non-zero element, return it.
    if (length(n) == 1) {
        result = n
    }
    # if there are exactly two values, check if they are duplicates.
    if (length(n) == 2) {
        result = lookup_function_duplicate(n)
    }
    # if there are exactly three values,check if they are triplicates.
    if (length(n) == 3) {
        result = lookup_function_triplicate(n)
    }
    # if there are more than three values, return -1.
    if (length(n) > 3) {
        result = -1
    }
    return(result)
}
```

Note about PPMs: parts per million (PPM) is a measure of mass error which is the difference between the exact mass (from the formula of the compound) and the accurate mass (from measured value on the mass spectrometer). This error when divided by the exact mass and mulitplied by 1e6 which gives us PPM.
For a given mass 'm', this function finds which masses in 'a' are within 'p' ppm of 'm' and returns the index of each. Both 'x' and 'a' must of the integer type.

```
filter_M <- function(m, a, p) {
    which(abs((m - a) * 1000000 / a) <= p) #it is a hit
}
```

For a given mass 'm', this funciton subtracts 1 Proton from it and then finds which masses in 'a' are within 'p' ppm of that.

```
filter_H <- function(m, a, p) {
    which(abs((m - PROTON - a) * 1000000 / a) <= p) #it is a hit
}
```

For a given mass 'x', this funciton subtracts the mass of 1 Sodium atom and 1 electron from it and then finds which masses in 'a' are within 'p' ppm of that.

```
filter_Na <- function(m, a, p) {
    which(abs((m - SODIUM_Plus - a) * 1000000 / a) <= p) #it is a hit
}
```

Data

```
f = as.data.frame(fread(input = bucket_table_name, header = T))
f.d = f[, 2:length(f)]  # Take only the numerical values
```

Add the row and column names

Note: As before, make sure to comment out the lines specific to the dataset you are evaluating.

```
#Specific pattern depending on naming convention
pattern = "[A-Z]\\d+" #For both datasets
# rownames(f.d) = str_extract(string = f[ ,1], pattern = pattern) # For
larger dataset
# rownames(f.d)[4:dim(f.d)[1]] = str_extract(string = f[4:dim(f)[1] ,1],
#                               pattern = pattern) # For smaller dataset
# rownames(f.d)[1:3] = c("B499", "A165", "A2171") # For smaller dataset
# Generic pattern for column names assuming data is in min not seconds
pattern = "(\\d+.\\d+)min : (\\d+.\\d+)m\\/z"
colnames(f.d) = str_replace(string = colnames(f)[2:length(f)],
                            pattern = pattern,
                            replacement = "\\1_\\2")
```

Apply Pareto Scaling

```
f.d.scaled.pareto = as.data.frame(ParetoScale(f.d))
```

Run the PCA (scaling and centering has been done above)

```
fd.pca = prcomp(x = f.d.scaled.pareto, scale. = F, center = F)
```

The code to generate figures with different colors for different Euclidean Distances may be used at this point.

```
## Figure containing Loadings plot with color and graph showing how
quickly Euclidean Distance decreases in a typical loadings plot.
d = fd.pca$rotation[ ,1:2]
d = as.data.frame(add_euclidean_distance(d))
d = d[order(d$`Euclidean Distance`, decreasing = T), ]
d$col = rep(c("red", "blue", "green", "orange"),
            times = c(2000, 2000, 2000, dim(d)[1] - 6000))
tiff(
    filename = "Colored-Loadings-smaller-dataset.tiff",
    width = 12,
    height = 6,
    units = "in",
    res = 300
     )
par(mfrow = c(1, 2))
plot(
    x = d$PC1,
    y = d$PC2,
    pch = 16,
    col = d$col,
    xlab = "PC1",
    ylab = "PC2",
    main = "Loadings Plot"
)
plot(
    x = d$`Euclidean Distance`,
    pch = 16,
    col = d$col,
    xlab = "Index",
    ylab = "Euclidean Distance",
    main = "Euclidean Distance vs Index"
)
dev.off()
```

Highest PCs

To figure out which strains stick out in which PCs, we need to sort them by the PC in which they are the highest - essentially mimicking the "scores overview" plot from Bruker's ProfileAnalysis.
The second line removes the magnitude of the index values because they are unimportant. All we need to know is which PCs are the highest and in what order. There is an additional step of removing "ix" from the column names.

```
fd.highestPCs = as.data.frame(apply(
    X = abs(fd.pca$x),
    MARGIN = 1,
    FUN = function(i)
        fd.sort(i)
))
fd.highestPCs = fd.highestPCs[, which(str_detect(string =
colnames(fd.highestPCs),
                                                 pattern = ".ix"))]
colnames(fd.highestPCs) = str_replace(
    string = colnames(fd.highestPCs),
    pattern = ".ix",
    replacement = ""
)
## To get pareto scaled data back scores %*% t(loadings)
#invisible(fd.pca$x %*% t(fd.pca$rotation)) #invisible hides output
```

Get unique masses

```
fd.unique = apply(
    X = f.d,
    MARGIN = 2,
    FUN = function(x)
        different_function(x)
)
## Fish out the -1s and return everything else's column index
filter_uniquemass = as.integer(which(fd.unique != -1))
```

If we apply the Unique masses filter to the bucket table, and then from that, we filter out the columns which are zero, we can get the unique masses for THAT row (strain). Note: the zero filtering step happens later, for now we simply take the masses which are unique to a strain from the bucket table.

```
buckets_unique = f.d[, filter_uniquemass]
```

Antibase

We read the antibase database and cleanup the column containing exact masses and the compound names.

```
if (FLAG == 1) {
    # Read antibase
    antibase <- read.csv("~/Lab/antibase_tableout.csv",
                         stringsAsFactors = FALSE)
    # Take exact mass column
    antibase.exactmass = antibase$StructCalc
```

```
    # Cleanup: finds the first space and deletes it and
    # everything after it leaving only the exact mass.
    antibase.exactmass = str_replace(string = antibase.exactmass,
                                     pattern = " .*",
                                     replacement = "")
    # Make it of numeric type. This causes
    # an 'NAs introduced by coercion' warning. This is ok because we want
    # all non numeric values to be NA and can convert them to 0 later
    antibase.exactmass = as.numeric(antibase.exactmass)
    antibase.exactmass[which(is.na(antibase.exactmass))] = 0
    # Fill in blanks in the 'Names' column
    antibase$Name[which(antibase$Name == "")] = "NO_NAME"
}
```

Excel sheet initialization. The name is the same as the name of the bucket table.
Note: this assumes there is no excel sheet of that name in the folder being executed.

```
wb = createWorkbook()
saveWorkbook(wb, paste(bucket_table_name, ".xlsx", sep = ""))
```

Final Program

Takes row number 'r' from the modified bucket table and outputs the unique masses ordered by their euclidean distance on the loadings plot for that strain. Also outputs list of AntiBase matches and non-matches into an Excel spreadsheet based on user set FLAG at the beginning of the script.
Note z,d are temporary variables for the loop.

```
for (r in 1) {
    d = fd.pca$rotation[, fd.highestPCs[1:2, r]] #table containing PC
planes of the strain in question
    d = as.data.frame(add_euclidean_distance(d)) #add euclidean distance
    d$m = str_replace(
        string = rownames(d),
        pattern = ".*_",
        replacement = "") # add column of masses to serve as a key
    z = as.data.frame(t(buckets_unique[r, ])) # masses for that strain
    z$rt = str_replace(
        string = rownames(z),
        pattern = "_.*",
        replacement = ""
    ) # add a time column
    z$m = str_replace(
        string = rownames(z),
        pattern = ".*_",
        replacement = "") # Add mass column to serve as a key
    z = as.data.frame(z[z != 0, ]) # remove zeroes - only unique strain
masses left
    zd = merge(x = z, y = d, by.x = 'm', by.y = 'm', sort = F) # merge on
keys
    zd = zd[order(zd$`Euclidean Distance`, decreasing = T), ] # sort by
Euclidean distance
    colnames(zd)[2] = "Intensity"
    zd = zd[, c(3, 1, 2, 4, 5, 6)]
```

```
    if (dim(zd)[1] >= 10000) {
        zd = zd[1:10000, ]
    }
    if (FLAG == 1) {
        # Now search for this mass list in antibase, and for each hit,
        # report the name of the hit.
        mzrt = read.table(text = rownames(z),
                          sep = "_",
                          colClasses = "numeric")
        z$rt = mzrt$V1
        z$mz = mzrt$V2
        # Column for putting in (later) which masses are NOT in AntiBase
        z$Unique = NA
        # Search in AntiBase
        match_mass = lapply(z$mz, function(x)
            filter_M(x, antibase.exactmass, ppm))
        match_hydrogen = lapply(z$mz, function(x)
            filter_H(x, antibase.exactmass, ppm))
        match_sodium = lapply(z$mz, function(x)
            filter_Na(x, antibase.exactmass, ppm))
        rows_for_mass = sum(unlist(lapply(match_mass, function(x)
            length(x))))
        rows_for_hydrogen = sum(unlist(lapply(match_hydrogen, function(x)
            length(x))))
        rows_for_sodium = sum(unlist(lapply(match_sodium, function(x)
            length(x))))
        #Finds how many matches were found for all of the masses across
M, H, Na
        totalrowsneeded = sum(rows_for_mass, rows_for_sodium,
rows_for_hydrogen)

        # Next, we want to filter out the masses from the 'match_mass'
which are hits in AntiBase. It is a list of lists, where the upper level
is the index in the 'match_mass' and lower number is the index in the
antibase.exactmass list.
        # Excel sheet preparations
        wb = loadWorkbook(file = "./1.xlsx")
        # make sheet = strain name
        sheet = createSheet(wb,
                            sheetName = rownames(buckets_unique)[r])
        # make headers for the row
        firstRow = matrix(data = c("RT_MZ",
                                   "M Match",
                                   "M--H Match",
                                   "M--Na Match"),
                          nrow = 1)
        # define a cell block
        cb = CellBlock(
            sheet = sheet,
            startRow = 1,
            startColumn = 1,
            noRows = 1,
            noColumns = 4,
            create = T
        )
```

```
        # put the headers there
        CB.setMatrixData(
            cellBlock = cb,
            x = firstRow,
            startRow = 1,
            startColumn = 1,
            showNA = T
        )
        # an index to keep track of where to put the next mass being
matched
        previousEnd = 2
        for (i in 1:dim(mzrt)[1]) {
            cb = CellBlock(
                sheet = sheet,
                startRow = previousEnd,
                startColumn = 1,
                noRows = 1,
                noColumns = 1,
                create = T
            )
            CB.setMatrixData(
                cellBlock = cb,
                x = as.matrix(rownames(z)[i]),
                startRow = 1,
                startColumn = 1,
                showNA = T
            )
            # define cell block for M, H, Na matches and add data to the
block
            # For Mass matches
            if (length(match_mass[[i]]) != 0) {
                massdata = as.matrix(antibase$Name[match_mass[[i]]])
                numberofRowsM = length(match_mass[[i]])
                cb = CellBlock(
                    sheet = sheet,
                    startRow = previousEnd,
                    startColumn = 2,
                    noRows = numberofRowsM,
                    noColumns = 1,
                    create = T
                )
                CB.setMatrixData(
                    cellBlock = cb,
                    x = massdata,
                    startRow = 1,
                    startColumn = 1,
                    showNA = T
                ) # write the data
            } else {
                numberofRowsM = 0
            }
            # For Hydrogen Matches
            if (length(match_hydrogen[[i]]) != 0) {
                hydrogendata =
as.matrix(antibase$Name[match_hydrogen[[i]]])
```

```
        numberofRowsH = length(match_hydrogen[[i]])
        cb = CellBlock(
            sheet = sheet,
            startRow = previousEnd,
            startColumn = 3,
            noRows = numberofRowsH,
            noColumns = 1,
            create = T
        )
        CB.setMatrixData(
            cellBlock = cb,
            x = hydrogendata,
            startRow = 1,
            startColumn = 1,
            showNA = T
        ) # write the data
    } else {
        numberofRowsH = 0
    }
    # For Sodium Matches
    if (length(match_sodium[[i]]) != 0) {
        sodiumdata = as.matrix(antibase$Name[match_sodium[[i]]])
        numberofRowsNa = length(match_sodium[[i]])
        cb = CellBlock(
            sheet = sheet,
            startRow = previousEnd,
            startColumn = 4,
            noRows = length(match_sodium[[i]]),
            noColumns = 1,
            create = T
        )
        CB.setMatrixData(
            cellBlock = cb,
            x = sodiumdata,
            startRow = 1,
            startColumn = 1,
            showNA = T
        ) # write the data
    } else {
        numberofRowsNa = 0
    }
    if (max(numberofRowsNa, numberofRowsH, numberofRowsM) != 0) {
        previousEnd = previousEnd +
            (max(numberofRowsNa, numberofRowsH, numberofRowsM))
    } else {
        cb = CellBlock(
            sheet = sheet,
            startRow = previousEnd,
            startColumn = 2,
            noRows = 1,
            noColumns = 1,
            create = T
        ) # cell block of 1 cell
        CB.setMatrixData(
            cellBlock = cb,
```

```
                    x = matrix(c("NO MATCHES FOUND"), nrow = 1),
                    startRow = 1,
                    startColumn = 1,
                    showNA = T
                ) # say no matches found
                z$Unique[i] = 1
                previousEnd = previousEnd + 1
            }
        }
    }
    # Write the matrix 'z' as a data frame to the sheet for the current
strain. This is the list of masses sorted in order of euclidean distance
on the loadings plot. The last column includes a '1' for unique and a
blank for something found in AntiBase.
    if (FLAG == 0) {
        # Excel sheet preparations
        wb = loadWorkbook(file = paste(bucket_table_name, ".xlsx", sep =
""))
        # make sheet = strain name
        sheet = createSheet(wb,
                            sheetName = rownames(buckets_unique)[r])
    }
    addDataFrame(
        x = as.data.frame(zd),
        sheet = sheet,
        col.names = T,
        row.names = F,
        startRow = 1,
        startColumn = 6,
        showNA = F
    )
    saveWorkbook(wb, file = paste(bucket_table_name, ".xlsx", sep = ""))
}
```
